# One size doesn’t fit all: regional dynamics in pediatric emergency visits during the SARS-CoV-2 pandemic

**DOI:** 10.3389/fpubh.2025.1574208

**Published:** 2025-08-14

**Authors:** Michelle Seiler, Gregory Biland, Oliver Gruebner, Sergio Manzano, Gianluca Gualco, Marc Sidler, Ursula Laasner, Julia Dratva, Michael von Rhein

**Affiliations:** ^1^University Children’s Hospital Zurich, University of Zurich, Zurich, Switzerland; ^2^Department of Geography, University of Zurich, Zurich, Switzerland; ^3^Faculty of Health Sciences and Medicine, University of Lucerne, Lucerne, Switzerland; ^4^Department of Pediatric Emergency Medicine, Geneva Children’s Hospital, Geneva University Hospitals, and Faculty of Medicine, University of Geneva, Geneva, Switzerland; ^5^Department of Pediatric Emergency, Institute of Pediatrics of Southern Switzerland, Bellinzona, Switzerland; ^6^Professional Association of Swiss Pediatricians in Private Practice, Dietikon, Switzerland; ^7^Professional Association of Swiss Pediatricians in Private Practice, Winterthur, Switzerland; ^8^School of Health Science, ZHAW Zurich University of Applied Sciences, Winterthur, Switzerland; ^9^Medical faculty, University of Basel, Basel, Switzerland

**Keywords:** regional dynamics, pediatric, emergency departments, SARS-CoV-2 pandemic, health geography

## Abstract

**Background and aims:**

The Swiss government implemented lockdown measures during the COVID-19 pandemic to contain outbreaks and prevent healthcare system overload. Emergency department (ED) visits were discouraged, leading to a decline in utilization, except for urgent cases. However, little is known about regional variations in pediatric ED use and spatial distribution patterns across Switzerland. This study aimed to analyze changes in pediatric ED catchment areas over time across three Swiss centers and explore implications for future healthcare crises.

**Methods:**

We conducted a retrospective, longitudinal cohort study at three tertiary pediatric EDs in Zurich, Bellinzona, and Geneva, covering different language regions. Data from March 2018 to February 2022 included daily pediatric ED visits, patient demographics, and postal codes. We categorized the timeline into pre-pandemic (before March 11, 2020), pandemic (March 11, 2020 – March 22, 2021), and post-pandemic (after March 22, 2021) phases. Travel distances were analyzed using driving distance matrices along the Swiss road network, and geovisualization techniques were applied to illustrate regional variations and policy impacts.

**Results:**

Among 294,409 recorded ED visits (158,643 in Zurich, 32,332 in Bellinzona, 103,434 in Geneva), ED visits declined by approximately 50% during the lockdown. Before the pandemic, patients living closer to hospitals visited more frequently. During the pandemic, the decline was most pronounced among nearby residents, while post-pandemic utilization shifted toward patients living farther away. Regional differences were observed: travel distances remained stable in Zurich; in Bellinzona, they increased by nearly 11%; and decreased by 3% in Geneva.

**Conclusion:**

The pandemic significantly influenced pediatric ED utilization in Switzerland, with long-term shifts in healthcare-seeking behavior. Despite uniform national regulations, utilization patterns varied across language regions, suggesting multifactorial influences. Geographic visualization provided insights into catchment area changes, offering a valuable tool for healthcare planning. These findings highlight the need for region-specific strategies in future healthcare crises, and our approach can be applied to other Swiss regions and similar global settings.

## Background

The COVID-19 pandemic prompted a range of public health containment and control measures, including social distancing, hygiene measures, and restrictions on public gatherings, to contain local outbreaks and avoid an overload of healthcare systems (1–3). To prevent overcrowding in emergency departments (EDs), their use was discouraged and dropped accordingly. Exceptions for patients requiring immediate medical attention were allowed. However, regarding children, less is known about understanding heterogeneity in the change in health service change across regions and patient catchment areas of pediatric EDs throughout the pandemic. Nevertheless, pediatric healthcare significantly changed during the initial phases of the pandemic. Many countries reported marked decreases in pediatric primary care and pediatric ED visits (4–6), ranging from 57% in Canada to 88% in Italy (7–12). These declines were attributed to fear of COVID-19 exposure, reduced incidence of other illnesses (13, 14), and changes in healthcare-seeking behavior (15). Studies in pediatric healthcare utilization during the COVID-19 pandemic revealed significant disparities between urban and rural areas, with more declining ED visits in urban areas as compared to rural areas (16–18).

Despite their increasing recognition as valuable information in healthcare planning and analysis (19, 20), geographic information and analysis have only be minimally used to study service accessibility and dynamics in pediatric care provision during the pandemic. It has been explicitly called for more studies that combine spatial and non-spatial factors to optimize healthcare access, particularly in resource-limited populations (21). In addition, such approaches are particularly needed in the context of pediatric emergency care, where timely access is critical, and geospatial models of dynamic catchment areas can provide essential insights (22).

So far, only a few studies have used longitudinal analyses that account for the pre-pandemic period or extended across the pandemic phase (23–25). Furthermore, most longitudinal research was confined to data from the initial pandemic year without examining utilization patterns after lifting of the most stringent containment measures. While extensive research has explored the impact of COVID-19 and associated containment measures on pediatric healthcare utilization in many countries, studies focusing on Switzerland are limited.

In Switzerland, the government introduced stringent public health measures to reduce the transmission of COVID-19 and protect the healthcare system. Measures included school closures, restrictions on elective and nonemergent medical care, and a nationwide lockdown, initiated on March 16, 2020. These interventions were gradually eased with schools reopened on May 11, 2020, and elective and nonemergent medical care resuming on May 27, 2020. All restrictions were lifted entirely by April 2022. Despite uniform regulations across the country, prior research by our group described substantial regional differences in pediatric ED utilization dynamics across Switzerland’s three language regions (26). However, these analyses did not provide detailed insights into the spatial dynamics of healthcare utilization patterns or offer insights into catchment area shifts for pediatric ED visits.

Therefore, the objective of this study was to address these gaps by leveraging geographic visualization and analysis to explore catchment areas for three pediatric EDs in Switzerland spatially. We aimed to integrate readily available data that might be used in real time for future public health planning. Specifically, we investigated how the spatial patterns within the catchment areas of three Swiss EDs changed over time during the pandemic.

## Materials and methods

Data retrieval and data types used in this retrospective longitudinal observational study have already been described in our previous publication (26). In brief, we used data from three tertiary pediatric EDs in Switzerland, representing the main language regions, from March 1, 2018, to February 28, 2022. The participating EDs are the sole tertiary pediatric EDs in their respective regions: one in the northern, German-speaking part (Zurich, University Children’s Hospital), one in the western, French-speaking part (Geneva, University Hospital), and one in the southern, Italian-speaking part of Switzerland (Ticino, Pediatric Institute of Italian part of Switzerland). Their inclusion enables the exploration of potential cultural or organizational differences between language regions, as described by previous research (26). These EDs serve as the main referral institutions for pediatric emergency care within their regions and reflect local healthcare structures and utilization patterns.

Anonymous daily data were obtained for all ED patients during the study period, encompassing age (grouped into 0–4, 5–12, and 13–18 years), sex, triage category, hospital admission, nationality, and postal code of residence. Only patients with Swiss postal codes were included in the analysis.

The detailed statistical analysis was conducted using a structured methodology involving three steps: (1) data preparation and normalization, (2) cohort characterization and distribution, and (3) spatio-temporal visualization and analysis. In the first step, the dataset was segmented into three distinct periods to reflect different phases in the context of the pandemic: before (March 1, 2018 - March 10, 2020), during (March 11, 2020 - March 21, 2021), and after (March 22, 2021 - March 1, 2022) the most severe non-pharmacological interventions. This segmentation allows for a comparative analysis across these temporal phases. Additionally, the distinction between cantonal and non-cantonal emergency visits allowed for a differentiated analysis, enabling the identification of regional differences and the impact of COVID-19 on these areas. A systematic approach was employed to compute normalized counts of pediatric visits. Initially, geographic units (postal codes by Swisstopo^1^) were used to aggregate the raw visitation data. Distances were computed between each ED and the geometric centroid of the patient’s residential postal code area, as provided by Swisstopo. The aggregation process involved counting the number of pediatric visits per postal code. These counts were normalized to ensure comparability across different regions. This normalization process adjusted the counts based on the total number of children (age 0–18) in each postal code^2^, resulting in normalized visitation rates per 100 children. This adjustment is crucial for mitigating the effects of varying population densities and ensuring that the visitation rates are representative.

To analyze the dynamics of the catchment areas of the three pediatric EDs before, during, and after the lockdown, travel distances for ED visits were computed using a distance matrix, which provided point-to-point distances for each postal code along the Swiss road network. The distance matrix approach utilized Swiss traffic zones to compute the shortest driving distance on the Swiss road network, calculated by the Federal Office for Spatial Development from the ARE^3^. Travel distances were capped at 60 km to exclude outliers. This computation was crucial for understanding how changes in travel distances might have influenced visitation rates at the children’s hospitals, offering insights into accessibility and patient behavior during the pandemic. The distances above and below each city’s median were compared to the pre-pandemic period to analyze travel distance changes over the three periods. Statistical analysis using Kolmogorov–Smirnov tests was conducted to determine significant changes in travel distances. Additionally, mean travel distances were calculated for each ED and period to identify trends in patient accessibility and behavior. For the visualizations, a reference limit for normalized visitation rates was established across all time periods to maintain consistency and allow direct comparisons. Changes in visitation rates were categorized into five quantile-based groups to better capture regional variation, with colors assigned from a diverging palette to distinguish increases from decreases visually. For the reference limit, 100% was chosen for each city’s first period using the respective maximum values. We used R Version 4.4.2 and Python Version 3.13.1 along with their standard packages and adaptations for data preparation, normalization, and spatial computations ^4^.

## Results

Over the study period from March 2018 to February 2022, 294′409 ED visits were recorded for our study areas, with 158′643 in Zurich, 103′434 in Geneva, and 32′332 in Ticino. The median age of patients visiting the EDs was four years (inter-quartile range: 1–9 years), with 54.7% children up to four years old, 35.1% aged 5–12 years, and 10.2% aged 13–18 years. The rates of intra-cantonal and extra-cantonal places of residence of the patients treated before, during, and after the pandemic varied significantly (*p* < 0.05) between the three centers and periods, with Zurich treating 7.35% of patients with extra-cantonal origin before, 8.02% during, and 7.8% after the pandemic. In Bellinzona, the rates of patients with extra-cantonal residence were 5.98, 6.64, and 7.05% (*p* < 0.05), and in Geneva, the rates were 6.62, 6.25, and 6.22% (*p* < 0.05) respectively.

**Figure 1:**
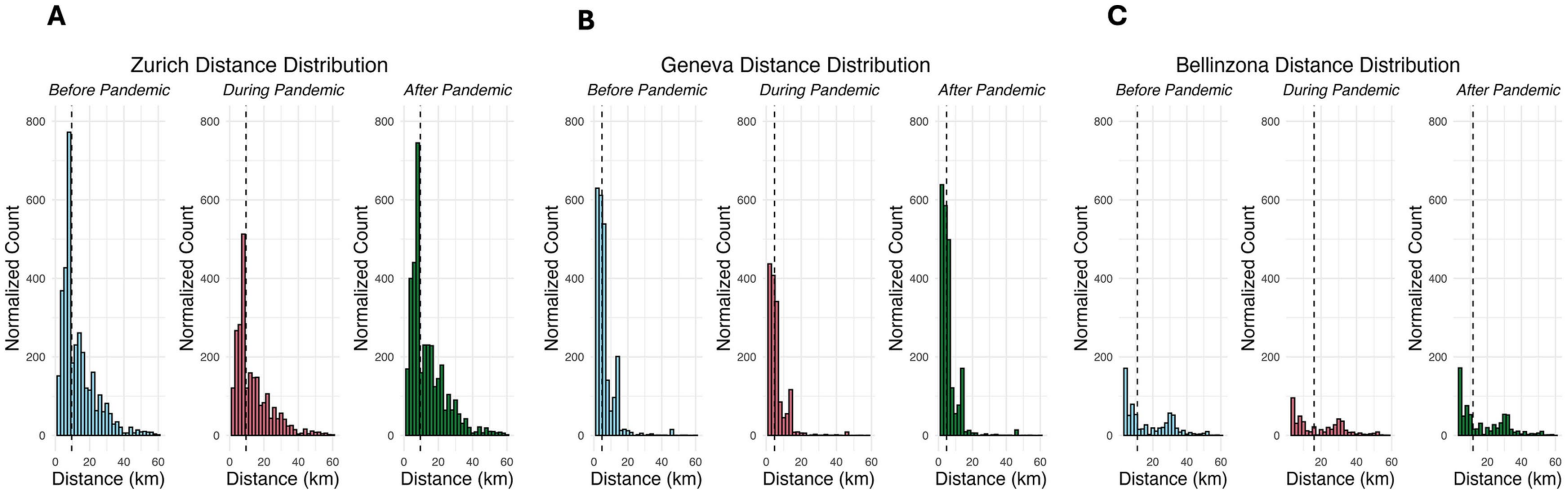
Distance distributions of computed travel distances for Zurich, Bellinzona, and Geneva during the three periods. Travel distances between residence and EDs in km before the pandemic, during the lockdown, and post-pandemic in **(A)** Zurich, **(B)** Geneva, and **(C)** Bellinzona. The plots are normalized, so the scales are identical for each city. Vertical dashed lines represent the median of the distance distribution.

The geographic analysis of travel distances to pediatric EDs during the COVID-19 pandemic also revealed statistically significant (*p* < 0.05) variations across different pandemic periods, reflecting changes in patient behavior and accessibility. In Zurich, the mean travel distance to the ED decreased slightly but significantly during the lockdown before significantly increasing after the lockdown. For Geneva, the mean travel distance substantially reduced during the lockdown, but no significant change was observed from the lockdown to the post-lockdown period. In Bellinzona, the mean travel distance significantly increased during the lockdown. Following the lockdown, there was a significant decrease in travel distances after restrictions were lifted (Table 1). However, despite the average minor differences in travel distance in Zurich between the three time periods, Figure 1 shows that a noticeable increase in the number of visits by children living near the ED massively influences the average toward a lower value. Taking the median in Figure 1 into account, it is noticeable that the distribution is heavily right-skewed for all three cities.

**Table 1:** Mean travel distances for Zurich, Bellinzona, and Geneva before, during, and after the pandemic.

	Before lockdown [km]	During lockdown [km]	Difference (%) (before-during)	*p*	After lockdown [km]	Difference (%) (during-after)	*p*	Difference (%) (before-after)	*p*
Zurich	14.887	14.876	−0.069	<0.001	15.067	1.280	<0.001	1.210	<0.001
Geneva	9.177	8.7199	−4.979	<0.001	8.897	2.033	n.s.	−3.047	<0.001
Bellinzona	23.153	27.113	17.101	<0.001	25.698	−5.217	<0.001	10.991	<0.001

The Kolmogorov–Smirnov test was used to assess the statistical significance of these changes.

The distribution of travel distances to the pediatric EDs remained largely consistent across all three time periods and centers, showing a stable right-skewed pattern throughout. In Zurich, the majority of visits before the pandemic came from areas closer to the hospital, with a right-skewed distribution indicating fewer visits from more distant locations. During the pandemic, the overall number of visits declined across all distance groups, but the reduction was more pronounced for those living farther away. Following the pandemic, visits increased again, particularly among those from more distant areas, although the overall distribution remained largely unchanged. In Bellinzona, the distribution of visits before the pandemic was already balanced between residents living closer and farther from the ED, with a consistent right skew. During the pandemic, visit numbers dropped across the board, with a slightly more pronounced decline for those living nearer to the hospital. Following the pandemic, the number of visits partially recovered, while the distance distribution remained unchanged. In Geneva, although most patients lived close to the hospital and the median distance was relatively low, the normalized distribution of visits remained right-skewed across all periods. Visit counts declined during the pandemic, with a more substantial drop among patients living closer to the ED. After the pandemic, visits from both groups increased, slightly shifting the distribution toward more distant areas, yet the overall shape remained skewed to the right.

**Figure 2:**
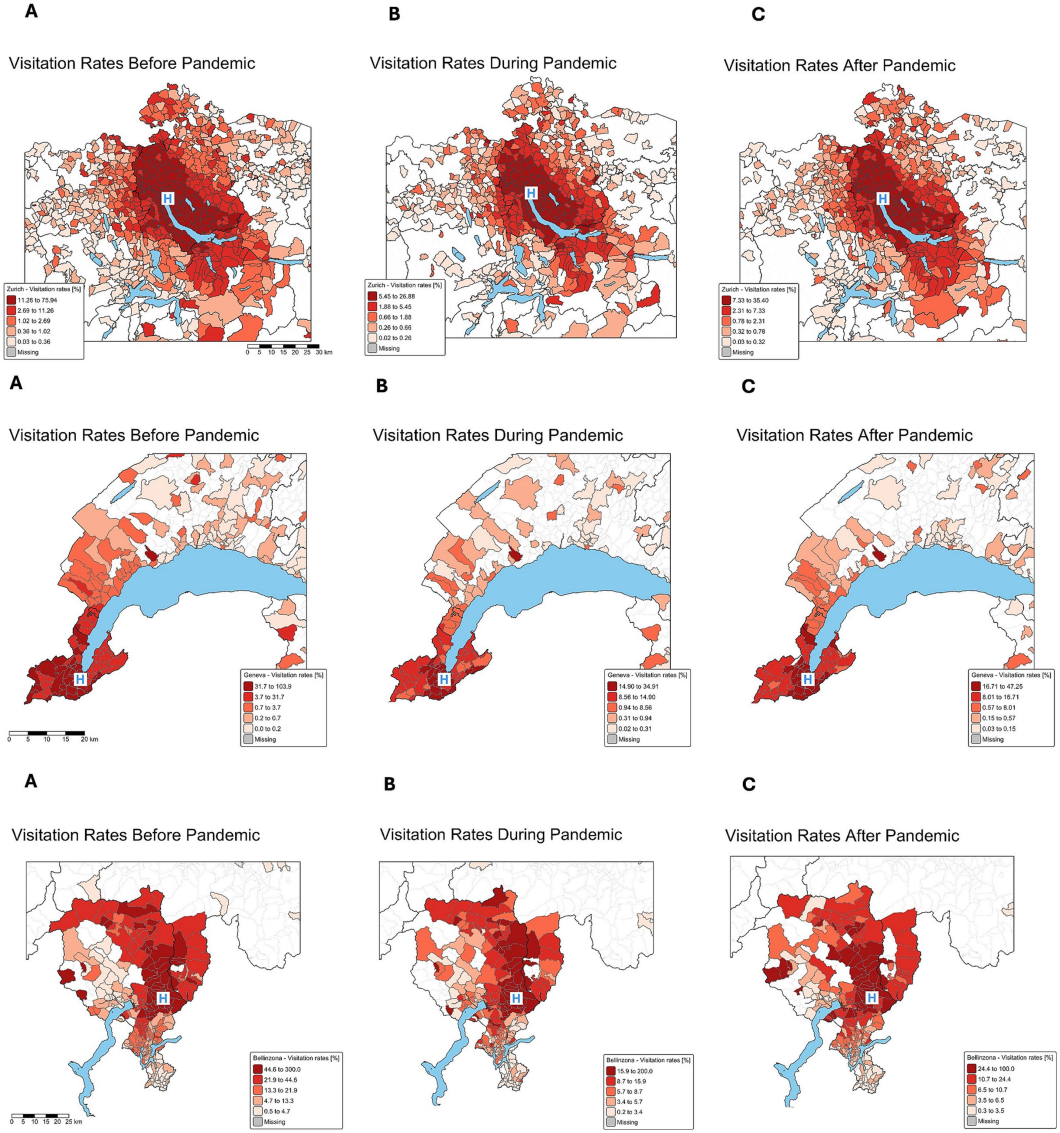
Spatial distribution of visitation rates during the three periods in Zurich, Geneva, and Bellinzona. Top row: Spatial distribution of normalized visitation rates [%] for Zurich, middle row: Geneva, bottom: Bellinzona for each period. Visitation rates are calculated based on the age-matched populations living in each postal code area. **A**: time period before the pandemic, **B**: during lockdown, **C**: post-pandemic period. Color-coded visitation rates represent the percentage of all children in each zip code who visited the ED. Blue “H” marks the hospital’s location.

The spatial distribution of visitation rates during the three periods is visualized in Figure 2. In Zurich, higher visitation rates were concentrated in regions closer to the hospital before the pandemic. During the pandemic, there was a significant decrease in visitation rates, particularly in the proximate regions of hospitals and a resurgence in visits post-pandemic, predominantly from more distant areas. Conversely, in Bellinzona, the normalized visitation rates for the Bellinzona ED across the three periods show that before the pandemic, visitation was more evenly distributed across the region before the pandemic, with only a slight concentration near the hospital. During the pandemic, Bellinzona experienced a more substantial drop in visitation rates, especially from nearby areas, and after the pandemic, there was a noticeable increase in visitation from more remote regions. In Geneva, higher visitation rates were observed in the hospital’s urban areas. During the lockdown, the population from these areas significantly reduced their visits. Post-pandemic, the rates rebounded and increased from more distant areas.

**Figure 3:**
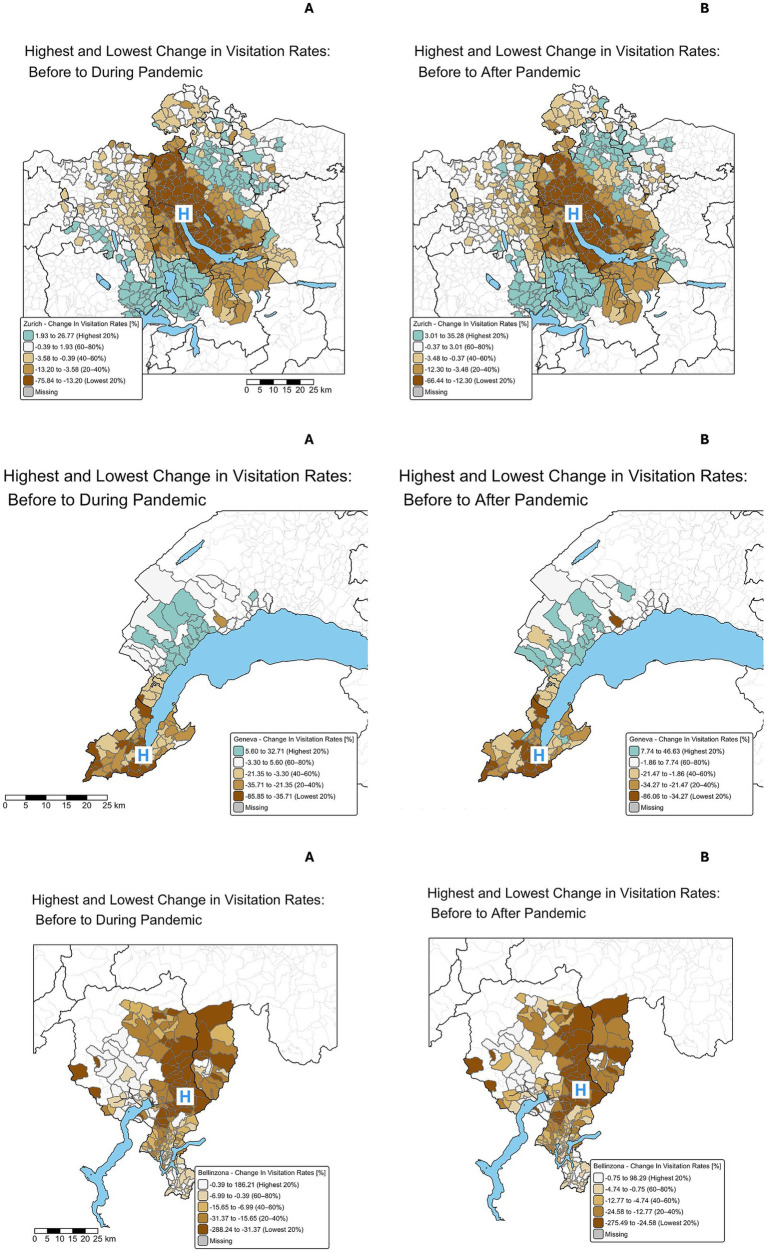
Spatial distribution of the changes of visitation rates to the ED for all three regions. Spatial distribution of the changes of normalized visitation rates [% change compared to prepandemic rate] to the ED for Zurich (top), Geneva (middle), and Bellinzona (bottom row) for the changes each before to during and before to after the pandemic. Relative change for each postal code area, referred to baseline, **A**: relative visitation rates before lockdown compared to pandemic, **B** relative visitation rates pre- vs. post-pandemic. Quintiles (n = 5) were calculated based on the distribution of percentage changes in visits per municipality. The lowest quintile (dark brown) represents the greatest reductions, and the highest quintile (blue-green) indicates the greatest increases. Blue “H” marks the hospital’s location.

The temporal changes in the visitation rates between the periods in the three regions are visualized in Figure 3. In Zurich, the reduction in visits during the lockdown compared to before the pandemic became more visible than in Figure 2. Visitation rates dropped up to 75% in areas close to the hospital. Post-pandemic, there was a notable increase of up to 25% in visits from more distant regions, compared to the pre-pandemic situation. In Bellinzona, a marked reduction in visits, especially from regions close to the ED, was observed, with reduction of visitations up to three times less, were as in the periphery the visitation rates locally doubled. In Geneva, the changes in the spatial distribution of normalized ED visitation rates before, during, and after the pandemic showed a significant decline in visitation rates from regions close to the hospital during the pandemic during the lockdown, with reduction in visitation rates up to 85%. In the post-pandemic phase, there was a noticeable rebound in visits, particularly from more distant areas with an increase up to 90%.

## Discussion

Our analyses of travel distances to pediatric EDs during the COVID-19 pandemic using the road network revealed statistically significant variations across different pandemic periods, reflecting changes in healthcare-seeking behavior and accessibility. In Zurich, the median travel distance to the pediatric ED only changed slightly, while Geneva and Bellinziona exhibited more pronounced changes. After the pandemic, average travel distances increased by almost 11% in Bellinzona but decreased by 3% in Geneva.

During the pandemic, global pediatric ED visits remarkably dropped in numerous countries, with decreases ranging from 50% in Canada and Switzerland to 88% in Italy (7–12, 26). This was primarily attributed to the stay-at-home policies and fear of catching or transmitting COVID-19 (7–10, 12, 24, 25, 27). However, our high-resolution analyses extend this understanding by highlighting regional differences in utilization patterns that remain undetected when focusing solely on visitor numbers. Specifically, utilization patterns changed differently in regions with different travel distances to the EDs during the pandemic, with the most noticeable drops observed in the immediate vicinity. Conversely, some more distant areas experienced increased visitation numbers, possibly attributable to disease severity or healthcare system modifications. For example, during the pandemic, certain adult hospitals were designated as COVID-19 hospitals, potentially influencing families to seek care at other EDs, even if they were farther away. Therefore, our geographic data demonstrate that the pandemic did not result in a scenario where only families living near these three hospitals were able to access the EDs. Families from more distant or rural areas also continued to seek care in EDs in Zurich, Geneva, and Bellinzona, despite the stay-home policy.

The normalized visitation rate maps provide further insight by visualizing regions of possible over- and underutilization. Before the pandemic, families living closer to the hospitals accounted for higher utilization, with rates decreasing with distance. During the pandemic, all three centers experienced fewer visits, particularly among those closer to the hospitals. The changes in healthcare-seeking behavior during the course of the pandemic and after can be recognized more clearly on the maps depicting the changes in the spatial distribution of the normalized visitation rates. Post-pandemic visits increased across all groups, particularly regarding visits from patients living farther away. This pattern suggests that the pandemic influenced healthcare-seeking behavior, leading to initial declines in ED visits, followed by a shift in utilization patterns, with increased reliance on ED services by those living farther from the hospitals. However, since the spatial distribution maps display the quintiles of the respective observed rates, the color coding visualizes the de facto utilization, and not the possible unmet need, or changes in utilization habits (i.e., choosing alternative healthcare services). However, complementary insurance data from the same period, discussed in separate paper of our group, confirm that these patterns reflect actual behavior rather than a shift to other service types (28).

The spatio-temporal changes of the visitation rates between the periods can be best acknowledged in Figure 3, which visualizes the significant reduction in visits during the pandemic compared to the period before, particularly from areas closer to the hospitals. The pediatric ED in Zurich experienced visitation drops up to 75%. The map underscores how the pandemic altered healthcare-seeking patterns, with a shift in reliance on ED services by those living farther from the centers, potentially reflecting organizational changes in the regional healthcare system, or decisions by families based on more subjective criteria. For instance, in Bellinzona and Geneva, there were structural changes of the area of responsibility of hospitals caring for COVID-19 cases. In the canton of Ticino, Locarno was designated to serve as facility solely for adult COVID-19 patients thus forcing more children to visit the ED in Bellinzona. However, such structural or organizational changes were not in place in the other two facilities. Other reasons why families living farther away increasingly accessed the ED during and after the pandemic (compared to regions of immediate vicinity) might include a perceived higher quality of care, changes in local healthcare services, including temporary reductions in the availability of outpatient pediatricians, or a subjective discomfort to visit primary care pediatricians for minor complaints or well-child-visits. For example, none of the pediatric EDs did extend their telemedicine services during the pandemic. However, some primary care pediatricians throughout the country provided remote consultations via phone or video, which may have temporarily altered care-seeking behavior. Such outpatient adaptations, although not systematically documented, could have influenced how and where families accessed urgent care during periods of restricted movement. This assumption is also supported by the finding, that the reduction was mostly observed in non severe cases (26).

In all three hospitals, post-pandemic utilization patterns differed from the pre-pandemic situation with relatively more patients from more distant areas, with distinct geographic patterns: In Zurich, patients residing in more distant areas to the north and south of the hospital were more likely to seek care during and after the pandemic. In contrast, a shift in patient flow was noted from east to west in Bellinzona, and toward the north in Geneva. These regional disparities suggest that, despite consistent regulations, healthcare utilization behavior can vary substantially, likely influenced by a range of factors such as regional differences in pandemic severity, local disease incidence, as well as cultural attitudes and habits. Besides distance, other factors such as travel time, severity of symptoms, prior provider relationships, or perceived risk may also have influenced ED attendance. These aspects should be explored in future studies for a more comprehensive understanding of care-seeking behavior during crises. Such cultural differences (29) have been shown to have an impact on the behavior of the population in different parts of Switzerland during the pandemic (i.e., mobility, social distancing) (30). This underscores the need for strategies that account for regional variations, rather than relying solely on broad, one-size-fits-all measures, in order to address large-scale health challenges effectively. They also emphasize the need for dynamic, real-time tools to monitor and visualize healthcare utilization patterns to better understand and respond to comparable events in the future. While our analysis focuses on spatial accessibility using travel distances, we acknowledge that other factors – such as perceived infection risk, availability of transportation, work flexibility, and broader social dynamics – likely played a role in shaping healthcare-seeking behavior during the pandemic. These multifaceted influences limit the extent to which conclusions can be drawn solely based on travel distance, but also based on travel time.

### Strengths and limitations

Our data show a change in healthcare-seeking behavior using travel distances. Even though possible reasons for the changes we found are discussed, it is noteworthy that due to limited data availability, we could not conduct analyses leading to a deeper understanding of possible causes. For instance, our analysis did not include changes in the operation of pediatric primary care during the pandemic. Although all participating EDs remained fully operational and continued to accept all patients, some primary care pediatricians in Switzerland individually turned to offering more remote consultations via telephone or video (28). This shift was very variable throughout the country as it was not forced by official restrictions, but rather by care seeking behavior of families, and the personal choices of primary care providers. Nevertheless, these shifts in outpatient care may have influenced ED utilization patterns and could partly explain the spatial changes we observed. However, the lack of systematic data on these changes limits our ability to assess their impact quantitatively. Further research is needed to investigate how service-level factors interact with spatial accessibility during different phases of the pandemic.

Additionally, while the three included EDs are the only tertiary pediatric EDs in their respective language regions, they differ in size, catchment areas, and annual patient visits. As such, utilization patterns observed in this study may be partially influenced by these institutional factors.

Our study is based on large datasets from three Swiss tertiary pediatric EDs representing the different language regions. However, the generalizability of the results to other pediatric hospitals in the respective language region as a whole may be limited as disparities of the populations served might exist. Unfortunately, due to data protection regulations in Switzerland, we were not able to perform additional analyses including socioeconomic background as a covariate, which might have provided an even deeper understanding as other groups found socioeconomic disparities to be a predictor of utilization changes (31). However, such additional variables can easily be added in future applications of our method. It is important to note that our analysis is based solely on Swiss data, and therefore, we cannot draw any conclusions regarding the effect of the pandemic on families living in neighboring countries and whether they continued to visit the EDs in Geneva and Bellinzona. Nevertheless, our methodological framework provides a robust approach for analyzing and visualizing changes in pediatric visitation rates over time and across different geographic regions, considering population normalization, geographic context, and travel distances.

Furthermore, our analysis focused on driving distances rather than actual travel times. While distance is a practical and widely used proxy for accessibility, it does not capture temporal variations such as changes in traffic conditions during lockdowns. Reduced congestion may have made longer distances more easily traversable, potentially influencing decisions about where to seek care. A more detailed analysis of travel times could provide further insight into accessibility, but such data were not available in our study.

## Conclusion

The COVID-19 pandemic had substantial effects on the number of pediatric ED visits, and influenced healthcare-seeking behavior of children and adolescents in Switzerland. Despite equal regulatory conditions, the utilization dynamics varied markedly between the three regions, highlighting the multifactorial modification of pediatric ED utilization during the pandemic. The observed effects outlasted the duration of the official containment measures, with increased reliance on ED services by those living farther from the hospitals. Our geographic visualization and analysis indicated spatial patterns within catchment areas over different pandemic periods. Our results illustrate the usefulness of such analyses for health crises measures and management, as well as in general. Variations between the different language regions suggest that uniform national measures may have varying effects depending on local context. Future management and policy decisions should therefore consider regionally tailored approaches, such as targeted communication or resource allocation, to better align with population behavior, especially under stress conditions.

Future geographically sensitive public health strategies should consider not only where people live, but how they access and interact with healthcare systems. Regional differences in mobility, healthcare infrastructure, and cultural norms must be accounted for to design effective, inclusive responses in future public health emergencies. The observed spatial redistribution of visits also suggests a need for real-time monitoring systems that integrate geographic, demographic, and clinical data to detect shifts in care-seeking patterns as they occur. Integrating such tools into regional health authority planning could enhance responsiveness during crises.

## Data Availability

The raw data supporting the conclusions of this article will be made available by the authors, without undue reservation.

## Footnotes

^1^ https://opendata.swiss/de/dataset/amtliches-ortschaftenverzeichnis-mit-postleitzahl-und-perimeter

^2^ https://www.bfs.admin.ch/asset/de/su-d-01.02.03.07

^3^ https://opendata.swiss/de/dataset/reisezeit-und-distanz-2017

^4^The entire code is available via a GitHub repository (https://github.com/grejonbil/pedCov-Analysis-KISPI.git).
